# Circadian transcription factor HSF1 regulates differential *HSP70* gene transcription during the arousal-torpor cycle in mammalian hibernation

**DOI:** 10.1038/s41598-018-37022-7

**Published:** 2019-01-29

**Authors:** Daisuke Tsukamoto, Tomoko Hasegawa, Shin-ichi Hirose, Yukina Sakurai, Michihiko Ito, Nobuhiko Takamatsu

**Affiliations:** Laboratory of Molecular Biology, Department of Biosciences, School of Science, Kitasato University, 1-15-1 Kitasato, Minami-ku, Sagamihara, Kanagawa 252-0373 Japan

## Abstract

Mammalian hibernation is a seasonal phenomenon. The hibernation season consists of torpor periods with a reduced body temperature (Tb), interrupted by euthermic arousal periods (interbout arousal, IBA). The physiological changes associated with hibernation are assumed to be under genetic control. However, the molecular mechanisms that govern hibernation-associated gene regulation are still unclear. We found that *HSP70* transcription is upregulated in the liver of nonhibernating (summer-active) chipmunks compared with hibernating (winter-torpid) ones. In parallel, HSF1, the major transcription factor for *HSP70* expression, is abundant in the liver-cell nuclei of nonhibernating chipmunks, and disappears from the nuclei of hibernating ones. Moreover, during IBA, HSF1 reappears in the nuclei and drives *HSP70* transcription. In mouse liver, HSF1 is regulated by the daily Tb rhythm, and acts as a circadian transcription factor. Taken together, chipmunks similarly use the Tb rhythm to regulate gene expression via HSF1 during the torpor-arousal cycle in the hibernation season.

## Introduction

Most mammals are homeothermic and maintain their body temperatures (Tb) within a narrow range despite variations in the ambient temperature. However, certain small mammals can undergo deep torpor, or hibernation, during the winter phase of their annual cycle. During the hibernation season, they enter repeated bouts of deep torpor by lowering their Tb to near 0 °C. Their heart and breathing rates also fall, and their metabolic rate is reduced to only a few percent of the euthermic level, enabling a considerable conservation of energy^1^. Since the discovery of a circannual hibernation rhythm in the golden-mantled ground squirrel in 1957^2,3^, endogenous circannual rhythms have been reported in several hibernating species of the squirrel family, including the chipmunk (*Tamias asiaticus*) used in this study^4^. The physiological changes associated with hibernation are assumed to be genetically regulated under the control of an endogenous circannual rhythm. Thus, elucidating the molecular mechanisms that control hibernation-associated gene regulation should shed light on the endogenous circannual process that dictates mammalian hibernation. Owing to recent advances in screening techniques, various differential gene expressions between nonhibernating (summer-active) and hibernating (winter-torpid) animals have been discovered, although the vast majority of genes show no change in transcript levels^5^. In addition, a failure to find genes that are unique to hibernating species supports the notion that the differential expression of mammalian genes leads to hibernation phenotypes^6^. On the other hand, the molecular mechanisms underlying the hibernation-associated gene regulation remain largely unexplored.

We recently showed that the differential transcription of the chipmunk *HP-25* gene between nonhibernating and hibernating chipmunks is regulated epigenetically^7^. The *HP-25* gene is expressed specifically in the liver, and is upregulated in nonhibernating chipmunks^8^. HNF-4 activates the liver-specific *HP-25* gene transcription^9^. Chromatin immunoprecipitation (ChIP) analyses revealed that H3K9 and K14 are highly acetylated and H3K4 is highly trimethylated in the *HP-25* gene promoter in the liver of nonhibernating but not hibernating chipmunks, and that significantly less HNF-4 binds to the *HP-25* gene promoter in hibernating chipmunks than in nonhibernating ones. Thus, there is a positive correlation between histone acetylation/trimethylation levels and the amount of HNF-4 bound to the *HP-25* gene promoter. We also observed that the *SHP* gene expression is upregulated in the liver of hibernating chipmunks, and that overexpressing SHP in primary hepatocytes prepared from nonhibernating chipmunks decreases the *HP-25* mRNA level, indicating that SHP is also involved in the hibernation-associated *HP-25* gene transcription^7^.

Here, to further elucidate the hibernation-associated gene regulation mechanisms, we searched for genes that are regulated in association with hibernation by subtractive cDNA cloning, and found that the *HSP70* mRNA is much more abundant in the liver of nonhibernating (summer-active) than hibernating (winter-torpid) chipmunks. HSP70 is the founding member of the highly conserved 70-kDa heat shock protein family of molecular chaperones^10^. HSP70 is normally maintained at low levels in cells, but it is induced under protein-damaging conditions, such as heat shock, oxidative stress, hypoxia, or heavy metals, and functions to provide resistance to a variety of proteotoxic stresses^11^. We further revealed that HSF1 is responsible for the transcriptional activation of the *HSP70* gene, and that the HSF1 activity is regulated by Tb rhythms, both during the wake-sleep cycle in the nonhibernation season and during the torpor-arousal cycle in the hibernation season. Our findings suggest that Tb rhythms strongly impact gene regulation during mammalian hibernation.

## Results

### *HSP70* transcription is activated in the liver of nonhibernating chipmunks

**Figure 1:**
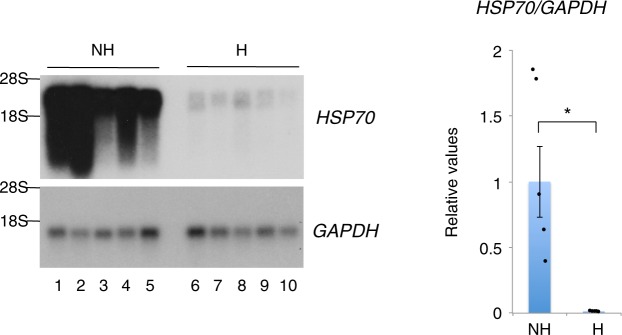
Hibernation-associated gene regulation of the *HSP70* gene. The *HSP70* and *GAPDH* mRNA levels were analyzed using poly(A)^+^-RNA prepared from the liver of nonhibernating chipmunks (ZT4-9) (NH: lanes 1–5) and hibernating chipmunks during a torpor bout (H: lanes 6–10) by northern blot analysis (left) and RT-qPCR (right). In the RT-qPCR analysis, the *HSP70* mRNA levels were normalized to that of the *GAPDH* mRNA, and the results are shown relative to the value obtained for nonhibernating chipmunks (NH). The results are shown as means ± SEM with corresponding dot plots to observed values (n = 5), and means were compared using non-parametric tests (Wilcoxon rank sum test). *p < 0.01; Wilcoxon rank sum test. The cropped northern blot analysis images are derived from different membranes. Full-length blots are presented in Supplementary Fig. S13.

To investigate the molecular mechanisms underlying hibernation-associated gene regulation, we applied a subtractive cDNA cloning procedure that enables the identification of genes whose transcript levels change dramatically between two conditions, and searched for genes that were expressed differentially in the liver between nonhibernating (summer-active) and hibernating (winter-torpid) chipmunks. We obtained nonhibernation-specific liver cDNA that was concentrated by subtraction using biotin-avidin binding and phenol extraction^12^. We then used this cDNA to screen a chipmunk liver cDNA library, and several clones for the *HSP70* mRNA were isolated. Northern blot and RT-qPCR analyses of liver RNA confirmed that the *HSP70* mRNA was much more abundant in nonhibernating than in hibernating chipmunks, while the *GAPDH* mRNA level did not change (Fig. 1). This finding indicated that the *HSP70* gene is activated in nonhibernating chipmunks, and not in hibernating ones. Similar nonhibernation-specific *HSP70* transcription is also observed in the liver of the Golden-mantled ground squirrel^13^. Notably, in contrast to the dramatic change in *HSP70* mRNA, the HSP70 protein level in liver was almost the same between nonhibernating and hibernating chipmunks (Supplementary Fig. S1).

### *HSP70* transcription is activated by HSF1 in nonhibernating chipmunks

**Figure 2:**
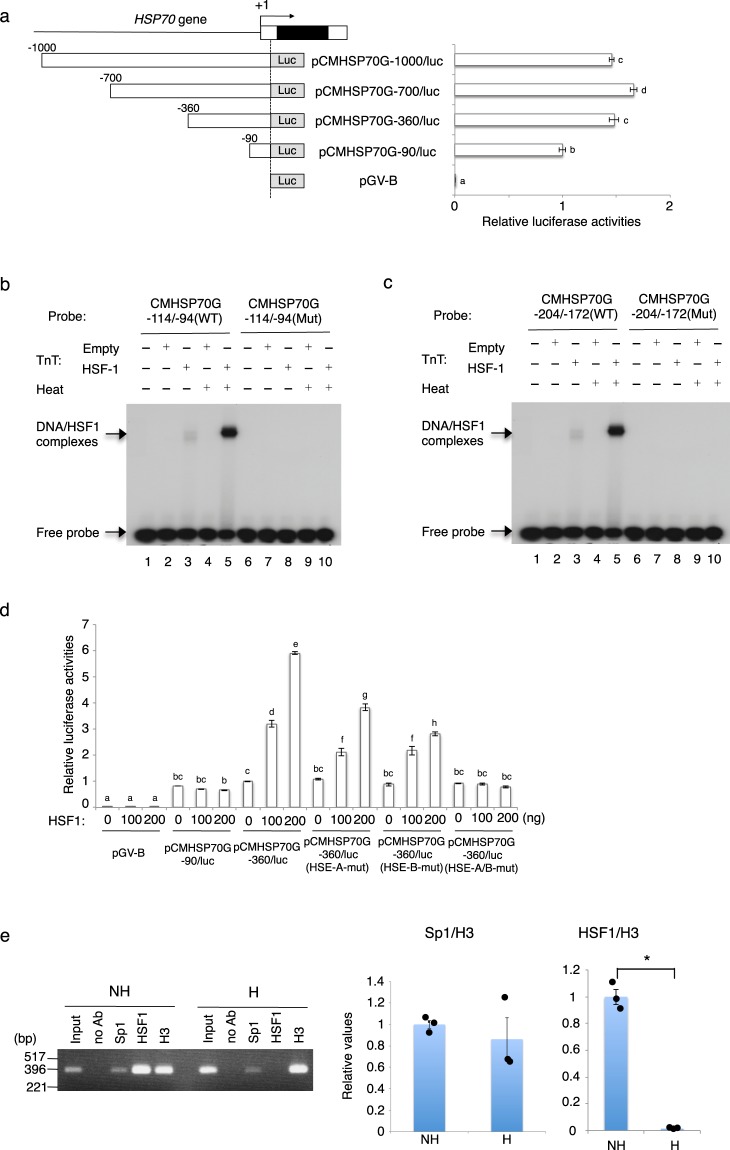
Promoter activity of the *HSP70* gene. (**a**) Schematic representation of the *HSP70* gene promoter. Deletion mutants were fused with the promoterless luciferase gene of pGV-B. Numbers indicate the position of the start site point of the fragment relative to the transcription start site. HepG2 cells were transfected with a promoter-reporter plasmid together with a *Renilla* luciferase plasmid pRL-SV40 as a control for transfection efficiency. Firefly luciferase activity was normalized to the *Renilla* luciferase activity and is shown relative to the value obtained with pCMHSP70G-90/luc. Data represent means ± SEM, and different letters (a–h) indicate significantly different values at p < 0.05; one-way ANOVA followed by Tukey test of a representative experiment performed in quadruplicate. Experiments were performed three times in all. (**b**) ^32^P-labeled CMHSP70G-114/-94(WT) (lanes 1–5) or CMHSP70G-114/-94(Mut) (lanes 6–10) was incubated with the *in vitro* transcription-translation products of pcDNA3 (lanes 2, 4, 7, and 9) or pcDNA3/mHSF1 (lanes 3, 5, 8, and 10). In lanes 4, 5, 9, and 10, the transcription-translation products were heat-treated at 42 °C for 60 min, and then incubated with the probe. (**c**) CMHSP70G-204/-172(WT) or CMHSP70G-204/-172(Mut) was used as a probe. (**d**) HepG2 cells were transfected with the indicated amounts of pcDNA3/mHSF1 together with one of the following *HSP70* gene promoter-reporter constructs: pCMHSP70G-90/luc, pCMHSP70G-360/luc, pCMHSP70G-360(HSE-A-Mut)/luc, pCMHSP70G-360(HSE-B-Mut)/luc, or pCMHSP70G-360(HSE-A/B-Mut)/luc, and pRL-SV40. The firefly luciferase activity was normalized to the *Renilla* luciferase activity, and the data are shown relative to the value obtained with pCMHSP70G-90/luc. Data represent means ± SEM, and different letters (**a**–**h**) indicate significantly different values at p < 0.05; two-way ANOVA with Tukey post hoc test of a representative experiment performed in quadruplicate. Experiments were performed three times in all. (**e**) ChIP (left panel) and ChIP-qPCR (right panel) were performed with chromatin from the liver of a nonhibernating chipmunks (ZT6-8) (NH) and a hibernating chipmunk during a torpor bout (H) using the indicated antibodies, normal rabbit IgG (IgG), or no antibody (no Ab). After DNA purification, the samples were analyzed by PCR using a primer set specific for the *HSP70* gene promoter. A portion of the total input sample was also examined by PCR. In the ChIP-qPCR analysis, the qPCR was performed in triplicate, and results are shown as means ± SEM with corresponding dot plots to observed values (n = 3). Values were normalized to the histone H3 value. Results are representative of experiments using chromatin from three each of nonhibernating and hibernating chipmunks. *p < 0.05; Welch’s *t* test. The cropped agarose gel image is derived from the same gel.

To analyze how *HSP70* gene transcription in the liver is regulated differentially between nonhibernating and hibernating chipmunks, we isolated genomic clones for the chipmunk *HSP70* gene and determined the gene structure. By 5′ RACE-PCR, we determined that the transcription start site was located 170-bp upstream of the translation initiation site ATG. The chipmunk *HSP70* gene was composed of a single exon, and TATA and CCAAT boxes were found 24- and 64-bp upstream of the transcription start site, respectively (Supplementary Fig. S2). We then constructed a series of promoter-luciferase reporter gene plasmids containing various lengths of the 5′ flanking region of the *HSP70* gene. These plasmids, together with a *Renilla* luciferase reporter plasmid pRL-SV40 as an internal control for transfection, were transfected into the human hepatoma cell line HepG2, and after 24 h, cell lysates were prepared and assayed for luciferase activity (Fig. 2a). The results revealed that the 90-bp 5′ flanking region of the *HSP70* gene contained the promoter for transcription in hepatocytes, and suggested that positively acting regulatory regions were present between −360 and −90. A database search of the 5′ flanking region using TFSEARCH^14^ revealed one Sp1-binding site (GC box) from −51 to −44 in this promoter region, and one and two consecutive HSF-binding sites (HSEs) from −109 to −98 (HSE-A) and from −199 to −178 (HSE-B) in the upstream region up to −360 (Supplementary Fig. S2).

We then tested whether Sp1 and HSF1 bound to these putative binding sites using electrophoretic mobility assays (EMSAs). *In vitro*-translated Sp1 bound to the double-stranded oligonucleotide CMHSP70G-62/-37(WT), which contained the sequence from –62 to –37, but not to the double-stranded oligonucleotide CMHSP70G-62/-37(Mut), in which the GC box sequence had two base substitutions (Supplementary Fig. S3). The double-stranded oligonucleotides CMHSP70G-114/-94(WT) and CMHSP70G-204/-172(WT) contained one HSE and two consecutive HSEs, respectively (Fig. 2b,c). When treated at 42 °C, *in vitro*-translated HSF1 bound to these WT oligonucleotides, but not to the same oligonucleotides containing two base substitutions in HSE (Mut) (Fig. 2b,c).

We next examined whether Sp1 could activate transcription from the *HSP70* gene promoter using the promoter-reporter plasmid pCMHSP70G-90/luc. When HepG2 cells were cotransfected with this reporter and a mammalian expression construct for Sp1, pCMHSP70G-90/luc showed increased luciferase activity in an Sp1 dose-dependent manner (Supplementary Fig. S4). On the other hand, pCMHSP70G-90(Sp1-mut)/luc, which carried the two base substitutions in the GC-box that abolished Sp1 binding (Supplementary Fig. S3), had only one-fourth the activity of pCMHSP70G-90/luc and was only weakly activated by Sp1, if at all (Supplementary Fig. S4). These results indicated that Sp1 binds to the GC-box in the *HSP70* gene promoter, and activates transcription from it. Similarly, in HepG2 cells, the transcription from pCMHSP70G-360/luc was activated by HSF1 in a dose-dependent manner (Fig. 2d). Mutations in either HSE-A or HSE-B partially inhibited the transactivation by HSF1, and when the HSEs in both HSE-A and HSE-B were mutated, no transactivation by HSF1 was observed (Fig. 2d), indicating that HSF1 activates transcription of the *HSP70* gene by binding to these HSEs.

To reveal whether Sp1 and HSF1 are involved in the transcriptional activation of the *HSP70* gene *in vivo*, we performed chromatin immunoprecipitation (ChIP) analyses using the liver of nonhibernating and hibernating chipmunks (Fig. 2e). ChIP revealed that Sp1 was bound to the *HSP70* gene promoter region in both nonhibernating and hibernating chipmunks, indicating that Sp1 is involved in *HSP70* gene transcription, but not in the hibernation-associated regulation of the *HSP70* gene. On the other hand, the amount of HSF1 bound to the *HSP70* gene promoter region was dramatically different between nonhibernating and hibernating chipmunks; it was greatly decreased in hibernating chipmunks (Fig. 2e), in agreement with the low *HSP70* mRNA level in these animals. These results suggested that HSF1 regulates *HSP70* gene transcription in a hibernation-associated manner.

### HSF1 activity is regulated by its shuttling between the nucleus and cytoplasm

**Figure 3:**
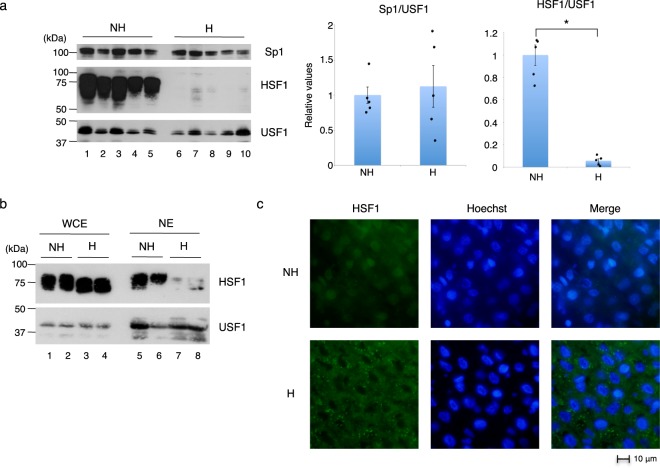
Involvement of HSF1 in *HSP70* gene transcription in the liver of nonhibernating chipmunks. (**a**) Immunoblot analysis of Sp1, HSF1, and USF1 performed using nuclear extracts prepared from the liver of nonhibernating chipmunks (ZT6-8) (NH) (lanes 1–5) and hibernating chipmunks during a torpor bout (H) (lanes 6–10). Quantified levels of Sp1 and HSF1 by ImageJ were normalized to that of USF1, and the results are shown relative to the value obtained for nonhibernating chipmunks (NH) (right panel). Results are shown as means ± SEM with corresponding dot plots to observed values (n = 5). *p < 0.05; Welch’s *t* test. The cropped immunoblot analysis images are derived from different membranes. Full-length blots are presented in Supplementary Fig. S14. (**b**) Immunoblot analysis of HSF1 and USF1 was performed using whole cell extracts (WCE) or nuclear extracts (NE) prepared from the liver of two each of nonhibernating chipmunks (ZT6-8) (NH) and hibernating chipmunks during a torpor bout (H). The cropped immunoblot analysis images are derived from different membranes. Full-length blots are presented in Supplementary Fig. S14. (**c**) Immunostaining with an anti-HSF1 antibody was performed on liver sections from a nonhibernating chipmunk (ZT6-8) (NH) and a hibernating chipmunk during a torpor bout (H), and observed by fluorescence microscopy. Nuclei were stained with Hoechst 33258.

To uncover how HSF1 is lost from the *HSP70* gene promoter in hibernating chipmunks, we compared the amounts of the transcription factors between nonhibernating and hibernating chipmunks by immunoblot analyses of liver nuclear extracts (Fig. 3a). In contrast to Sp1, whose levels were similar in the liver nuclei of nonhibernating and hibernating chipmunks, HSF1 was abundant in the liver nuclei of nonhibernating chipmunks, but almost disappeared in the nuclei of hibernating ones. On the other hand, the *HSF1* mRNA level in liver was almost the same between nonhibernating and hibernating chipmunks (Supplementary Fig. S5). Consistent with the mRNA level, similar amounts of HSF1 protein were detected in the whole cell extracts of nonhibernating versus hibernating chipmunks; however, the most slowly migrating species of HSF1 were found in nonhibernating chipmunks but not in hibernating ones (Fig. 3b, lanes 1–4). Immunohistochemical analysis confirmed the differential cellular localization of HSF1 in the liver between nonhibernating and hibernating chipmunks: HSF1 was localized mostly to the nucleus in nonhibernating chipmunks, but to the cytoplasm in hibernating ones (Fig. 3c and Supplementary Fig. S6a). These results indicated that the HSF1 activity is mainly regulated by nucleocytoplasmic shuttling in nonhibernating and hibernating chipmunks. Similarly, the nuclear HSF1 level was reduced in another peripheral tissue examined, the kidney, of hibernating chipmunks (Supplementary Fig. S6b).

**Figure 4:**
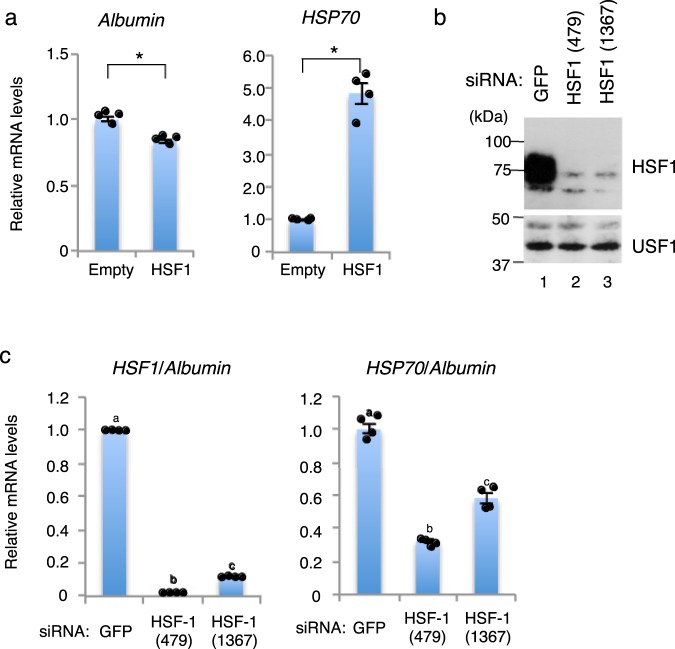
Effects of HSF1 on *HSP70* gene transcription in chipmunk primary hepatocytes. (**a**) Primary hepatocytes prepared from a hibernating chipmunk were transfected with pcDNA3 (Empty) or pcDNA3/mHSF1. Expression levels of the *HSP70* and *albumin* mRNA were measured by RT-qPCR. Data represent means ± SEM with corresponding dot plots to observed values (n = 4) of a representative experiment. Experiments were performed four times. *p < 0.01; Welch’s *t* test. (**b**,**c**) Primary hepatocytes prepared from a nonhibernating chipmunk were transfected with *HSF1* siRNA or *GFP* siRNA (negative control). (**b**) Knockdown of HSF1 by *HSF1* siRNA was confirmed by immunoblot analysis. The cropped immunoblot analysis images are derived from different membranes. Full-length blots are presented in Supplementary Fig. S15. (**c**) Expression levels of the *HSF1*, *HSP70*, and *albumin* mRNA were measured by RT-qPCR. The mRNA levels were normalized to that of the *albumin* mRNA. Data are expressed relative to the transcription level obtained with *GFP* siRNA. Data represent means ± SEM with corresponding dot plots to observed values (n = 4) of a representative experiment. Experiments were performed four times. Different letters (a–c) indicate significantly different values at p < 0.05; one-way ANOVA with Tukey post hoc test.

To elucidate whether HSF1 is involved in the transcriptional activation of the *HSP70* gene in chipmunk liver, we prepared primary hepatocytes from a hibernating chipmunk, and transfected them with a mammalian expression construct for HSF1. The overexpression of HSF1 in primary hepatocytes transfected with the HSF1 expression construct was confirmed by western blot analysis of nuclear extracts (Supplementary Fig. S7). As expected, HSF1 activated the *HSP70* gene transcription (Fig. 4a). We then performed a knockdown experiment with siRNA specific for the *HSF1* mRNA. For this analysis, primary hepatocytes were prepared from a nonhibernating chipmunk, and treated with siRNA targeting either *HSF1* or *GFP* (negative control). After 48 h, whole cell extracts and total RNA were prepared, and the knockdown of HSF1 by the *HSF1*-specific siRNAs was confirmed at both the protein and mRNA levels by immunoblot analysis and RT-qPCR, respectively (Fig. 4b,c). In contrast to the *GFP* siRNA, the two *HSF1*-specific siRNAs decreased the *HSP70* mRNA level (Fig. 4c). As expected from the small amounts of HSF1 expressed in the hepatocytes transfected with the *HSF1*-specific siRNAs, ChIP analyses revealed that the knockdown of HSF1 decreased the amount of HSF1 bound to the *HSP70* gene promoter (data not shown). These results indicated that HSF1 is involved in the transcriptional activation of the *HSP70* gene in chipmunk liver.

### HSF1 activity is regulated by body temperature (Tb) rhythm in the liver of nonhibernating chipmunks

**Figure 5:**
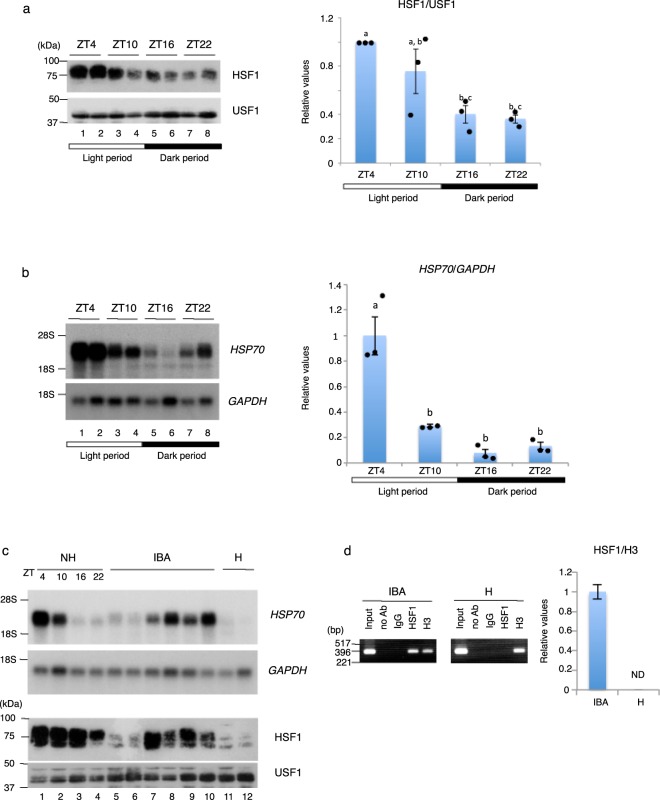
HSF1 activation by Tb rhythms. (**a**) Immunoblot analysis of HSF1 and USF1 using nuclear extracts prepared from the liver of nonhibernating chipmunks. HSF1 levels (Figs 5a and S9) quantified by ImageJ were normalized to those of USF1, and the results are shown relative to the value obtained at ZT4 (right panel). The chipmunks were kept at a 12 h:12 h light (white box):dark (black box) photoperiod (light on at 6 AM). Results are shown as means ± SEM with corresponding dot plots to observed values (n = 3). Different letters (a–c) indicate significantly different values at p < 0.05; one-way ANOVA with Tukey post hoc test. (**b**) Northern blot analysis and RT-qPCR of the *HSP70* and *GAPDH* mRNA were performed using liver poly(A)^+^-RNA. In the RT-qPCR analysis, the *HSP70* mRNA levels were normalized to those of the *GAPDH* mRNA, and the results are shown relative to the value obtained at ZT4. The chipmunks were kept at a 12 h:12 h light (white box):dark (black box) photoperiod (light on at 6 AM). Results are shown as means ± SEM with corresponding dot plots to observed values (n = 3 for each ZT including the two samples used for the Northern blot). Different letters (a,b) indicate significantly different values at p < 0.05; one-way ANOVA with Tukey post hoc test. (**c**) Northern blot analyses of the *HSP70* and *GAPDH* mRNAs were performed using liver poly(A)^+^-RNA (upper two panels), and immunoblot analyses of HSF1 and USF1 were performed using liver nuclear extracts (lower two panels). (**d**) ChIP (left panel) and ChIP-qPCR (right panel) were performed using the indicated antibodies, normal rabbit IgG (IgG), or no antibody (no Ab). After DNA purification, the samples were analyzed by PCR using a primer set specific for the *HSP70* gene promoter. In the ChIP-qPCR analysis, the qPCR was performed in quadruplicate, and results are shown as means ± SEM. Values were normalized to the histone H3 level. ND, not detectable. Experiments for interbout awake chipmunks were repeated using chromatin from three chipmunks. All of the cropped northern blot, immunoblot, and agarose gel analysis images are derived from different membranes or gels. Full-length blots are presented in Supplementary Fig. S16.

In mouse liver, although both the *HSF1* mRNA and total cellular HSF1 protein level are constant throughout the day, HSF1 is activated and accumulates in the nucleus during the nocturnal active phase, when Tb is elevated, and acts as a circadian transcription factor^15^. Chipmunks, a diurnal mammal, spend the nonhibernation season as a homeotherm like nonhibernators, and their Tb is high during the day and low during the night (Supplementary Fig. S8). We explored whether the HSF1 activity fluctuates daily in the liver of chipmunks in the nonhibernation season, by examining the nuclear HSF1 level by immunoblot analysis. As expected, the nuclear HSF1 level oscillated, increasing at around ZT4, at which time Tb is rising (Supplementary Fig. S8), in the active phase and decreasing at around ZT16, at which time Tb is declining (Supplementary Fig. S8), in the resting phase (Fig. 5a and Supplementary Fig. S9), although HSF1 was present in the nucleus throughout the day. In parallel, the *HSP70* mRNA level peaked at around ZT4 (Fig. 5b). These results also indicate that the large difference in the *HSP70* mRNA levels among the nonhibernating chipmunks observed in Fig. 1 would be due to the difference in sampling times. Similarly, the *HSP70* mRNA level is higher during the day than during the night in the liver of the Golden-mantled ground squirrel^13^. These results suggested that in the liver of nonhibernating chipmunks, the HSF1 activity is regulated by the daily Tb rhythm, and HSF1 is likely to act as a circadian transcription factor as is the case for mouse HSF1.

### HSF1 is activated by Tb rise during interbout arousal in the hibernation season

During the hibernation season, chipmunks are not continuously torpid: they arise for ~20 h at 5–7-day intervals, during which their Tb increases to the normothermic level of about 37 °C, and they subsequently return to hibernation and sustain a low Tb of 5–7 °C^16^. We therefore investigated whether the Tb rise during the interbout arousals activates HSF1 in the liver. The arousal process of the golden-mantled squirrel from torpor to euthermia lasts about 2 hr^17^, and chipmunks that were more than 2 hr after arousal showed the rectal temperature of 34–37 °C (Supplementary Fig. S10). We analyzed interbout awake chipmunks 2–6 hr after arousal from torpor, and found that in these animals, HSF1 accumulated in the nucleus (Fig. 5c, lanes 7–10). In parallel, in these interbout awake chipmunks, the *HSP70* mRNA level increased (Fig. 5c, lanes 7–10), and HSF1 was bound to the HSEs in the *HSP70* gene promoter (Fig. 5d). On the other hand, in the early-arousal phase, within one hour after arousal from torpor, the chipmunks’ rectal temperature was below 30 °C (Supplementary Fig. S10), and the amount of nuclear HSF1 was still low (Fig. 5c, lanes 5 and 6). Collectively, these results indicated that in the hibernation season, HSF1 is sequestered in the cytoplasm during torpor, but during interbout arousal, as the Tb rises, HSF1 is activated, accumulates in the nucleus, and activates *HSP70* gene transcription. These findings indicate that Tb rhythms have important roles in gene regulation both in the wake-sleep cycle of the nonhibernation season and in the torpor-arousal cycle of the hibernation season.

## Discussion

Mammalian hibernation is accompanied by various physiological changes that are assumed to be under genetic control. In this study, to examine the molecular mechanisms underlying hibernation-associated gene regulation, we analyzed how the *HSP70* gene in the liver is differentially regulated between nonhibernating (summer-active) and hibernating (winter-torpid) chipmunks. ChIP analyses revealed that HSF1 bound to the *HSP70* gene promoter in nonhibernating chipmunks, but not in hibernating ones, leading to the differential *HSP70* gene transcription. HSF1 was abundant in the nucleus in nonhibernating chipmunks, but excluded from the nucleus in hibernating ones. Further analyses revealed that HSF1 was regulated by Tb rhythms during the sleep-wake cycle of the nonhibernation season and during the torpor-arousal cycle of the hibernation season, and was activated in the wake and arousal phases when the Tb rises, leading to transcriptional activation of the *HSP70* gene.

In homeothermic mammals, the Tb still exhibits a daily fluctuation, falling during the rest phase and rising during the active phase^15^. In mouse liver, HSF1 activity is regulated by this Tb rhythm, and HSF1 acts as a circadian transcription factor^18^. The chipmunk is a diurnal mammal, and spends the nonhibernation season as a homeotherm. As expected, the nuclear HSF1 level in chipmunk liver showed a daily oscillation, peaking at ZT4 in the nonhibernation season (Fig. 5a). In parallel, the mRNA level of the *HSP70* gene, an HSF1 target gene, fluctuated daily with the zenith at ZT4 and the nadir at ZT16 (Fig. 5b), implying that HSF1 is activated around ZT4 and dampened around ZT16. However, in contrast to mouse, in which HSF1 disappears from the nucleus during the rest phase, HSF1 in chipmunks was present in the nucleus throughout the day (Fig. 5a). HSF1 activation can be divided into two separate steps: first, HSF1 forms a trimer, accumulates in the nucleus, and acquires DNA-binding activity^19^, and second, it acquires its transactivating capacity^20^. In parallel with this scenario, at ZT16 when the *HSP70* gene was only weakly transcribed (Fig. 5b), HSF1 still bound to the *HSP70* gene promoter (data not shown), showing that in the liver of nonhibernating chipmunks, HSF1 is mainly regulated at the step of acquiring its transactivating capacity. HSF1 is regulated exclusively at the post-transcriptional level including post-translational modification^21^. In fact, we found that the nuclear HSF1 migrated as a broad band ranging from 70 to 85 kDa, and that the most slowly migrating fraction of HSF1 was most abundant at ZT4 (Fig. 5a). When treated with bacterial alkaline phosphatase, HSF1 migrated as a single band with an approximate molecular weight of 65 kDa (Supplementary Fig. S11). Thus, inducible phosphorylations are likely to be involved in the circadian HSF1 activation. Budzynski *et al*. showed that HSF1’s transactivation activity is independent of its constitutive and stress-inducible phosphorylation within the regulatory domain, using a phosphorylation-deficient HSF1 mutant that lacks the known 15 phosphorylation sites within the regulatory domain^22^. Thus, the phosphorylations associated with HSF1’s circadian transactivation activity might occur outside the regulatory domain, or distinct combinations of phosphorylations might be involved, dependent on the stimulus.

We found that in contrast to the nonhibernation season, HSF1 activity in the chipmunk liver during the hibernation season is tightly regulated by nucleocytoplasmic shuttling, and HSF1 is activated during interbout arousal, resulting in *HSP70* gene transcription (Fig. 5c). In the absence of stress, most of the HSF1 exists diffusely distributed in the nucleus, due to a relatively potent import signal and a comparatively weak export activity^23^. Since HSF1 disappeared from the nucleus during torpor, HSF1’s import into the nucleus is likely to be inhibited during the entry into torpor. In the Arctic ground squirrel liver, several heat shock protein genes are overexpressed during the arousal from torpor^24^. These results collectively indicate that Tb rhythms play a crucial role in regulating gene expression, both during the sleep-wake cycle in the nonhibernation season and during the torpor-arousal cycle in the hibernation season, and that HSF1 is involved in this Tb rhythm-dependent gene regulation in both the nonhibernation and hibernation seasons. This notion is consistent with the observation that the daily sleep-wake cycle of the mouse and the torpor-arousal cycle of the Arctic ground squirrel in the hibernation season shared significant molecular signatures when their liver microarrays were compared^24^. In addition, even in interbout awake chipmunks, the amount of nuclear HSF1 was still low when the animals’ Tb was below 30 °C, indicating that HSF1 activation is likely to occur when the Tb rises above 30 °C (Fig. 5c, lanes 5, 6). Thus, despite the large Tb change from 6 °C to 37 °C in the hibernation season, a slight Tb change may be sufficient to activate HSF1, as in nonhibernators.

The daily Tb fluctuations act as systemic cues in the resetting of peripheral circadian clocks, and HSF1 is required for their efficient synchronization^19^. In this Tb-dependent entrainment of peripheral circadian clocks, HSF1 is supposed to act as an immediate early transcription factor, and activate *Per2* gene transcription^15,25^. Thus, our observation that HSF1 is activated during interbout arousal in the hibernation season implies a possibility that the peripheral circadian clocks may be reset during interbout arousal by Per2, although the persistence of circadian rhythms during hibernation remains controversial^26–28^. Also, considering the multifaceted roles of HSF1 in versatile physiological processes in eukaryotes^29^, analyses of HSF1 target genes might give a clue to understand the enigmatic role of the periodic interbout arousal in the hibernation season.

## Materials and Methods

### Animals

Male chipmunks (Tamias asiaticus) (age, 2–4 months) purchased from Pet Easy Space (Osaka) were individually housed and provided with standard rodent chow and water *ad libitum*. They were kept at 23 °C with a 12 h:12 h light:dark photoperiod (light on at 6 AM) during the nonhibernation season (April-September), and under a constant condition of 5 °C in darkness during the hibernation season (October-March). The conditions of hibernating chipmunks were monitored by an infrared activity sensor (O’Hara & Co., Ltd., Tokyo). Nonhibernating chipmunks (weight, 90–110 g; age, 1–3 years) were summer-active animals, and samples were obtained from them in June or July. Samples from hibernating chipmunks (during a torpor bout) (weight, 90–110 g; age, 1–3 years), whose rectal temperatures were 6–7 °C measured by a thermistor probe (Thermistor thermometer model KN-91-AD1687-R; Natsume Seisakusho, Tokyo), and interbout awake chipmunks were obtained approximately two to four months after the first entry into torpor between December and February, and the sampling time of hibernating chipmunks were 2–4 days after entry into deep torpor. Thus, all of the chipmunks were acclimated to the photoperiod and temperature condition of the nonhibernation or hibernation season for more than 2 months before the experimental samples were collected. Animals were sacrificed after being deeply anesthetized with isoflurane between 12 AM and 2 PM unless otherwise specified, and for Fig. 1, samples were collected between 10 AM and 3 PM. Tissues were immediately excised, frozen in liquid nitrogen, and stored at −80 °C until use. The Tb of interbout awake chipmunks was recorded by measuring the rectal temperature with a thermistor probe after anesthetization. All of the protocols were in accordance with the guidelines of the Institutional Animal Care and Use Committee of Kitasato University, and all experimental procedures were approved by the same committee.

### Cloning procedures

Oligo(dT)-primed double-stranded cDNA was synthesized from the liver poly(A)^+^-RNA of a nonhibernating (NH) chipmunk and a hibernating (H) chipmunk, and adaptors of different sequences were added to each NH and H cDNA. The H cDNA was then amplified by PCR using the adaptor sequence as a primer in the presence of biotin-11-dUTP. Subtractive cloning of nonhibernation-specific cDNA was carried out according to Duguid and Dinauer^12^ as follows. A mixture of 0.5 μg of NH cDNA and 10 μg of biotinylated H cDNA was denatured and then hybridized at 65 °C for 16 h. The biotinylated molecules were removed with streptavidin and phenol extraction, and then the resulting subtracted cDNA was ethanol precipitated. After three cycles of the hybridization/subtraction process, the subtracted DNA was amplified by PCR using the adaptor sequence as a primer, and used to screen a cDNA library constructed with NH cDNA.

Chipmunk *HSP70* genomic clones were isolated by screening a chipmunk genomic library using chipmunk *HSP70* cDNA as a probe as described^9^. The transcription start site of the chipmunk *HSP70* gene was determined by 5′ RACE-PCR using the First Choice RLM-RACE kit (Ambion).

### Northern blot analysis

Total RNA was prepared using ISOGEN (Nippon Gene), and poly(A)^+^-RNA was purified using Oligotex-dT30 (Takara Bio). Liver poly(A)^+^-RNAs from nonhibernating and hibernating chipmunks were fractionated by electrophoresis on a 1% agarose gel containing 2.2 M formaldehyde, and then transferred to a nylon filter. Hybridization was carried out using chipmunk cDNAs as probes as described^8^.

### Transfections and Luciferase Assays

HepG2 cells were cultured as described previously^9^. The promoter-reporter plasmids were constructed in the same way as described previously^9^. The cells were plated at 5 × 10^4^ cells per 15-mm dish, and after 24 h were transfected with 200 ng of a firefly luciferase promoter-reporter plasmid or a promoterless reporter plasmid, pGV-B, and 2.5 ng of a *Renilla* luciferase internal control plasmid, pRL-SV40 (Promega), using the TransIT-LT1 reagent (Mirus). Where denoted, the cells were cotransfected with the indicated amounts of the mouse HSF1 or Sp1 expression construct, pcDNA3/mHSF1 or pcDNA3/mSp1, respectively. Each transfection was carried out in quadruplicate. After 24 h, the luciferase activities were measured using the Dual-Luciferase Reporter Assay System (Promega). Firefly luciferase activity was normalized to the Renilla luciferase activity. The data are shown relative to the value obtained with pCMHSP70G-90/luc. Data represent means ± SEM of a representative experiment performed in quadruplicate. Statistics was calculated on individual experiments. Experiments were performed and confirmed three times in all.

### Electrophoretic mobility shift assay (EMSA)

Mouse Sp1 and HSF1 were synthesized using an *in vitro* transcription/translation system (Promega). Nuclear extracts from chipmunk liver were prepared as described^9^. The following oligonucleotides were annealed with complementary oligonucleotides and used as probes: CMHSP70G-62/-37(WT), 5′-GAGGAGGGAGCGGGCGGGGCTCCATG-3′; CMHSP70G-114/-94(WT), 5′-AACCCCTGGAACATTCCGGTC-3′; CMHSP70G-204/-172(WT), 5′-GAACCCCAGAAGCGTCTGGAGAGTTCTGGGGAG-3′; CMHSP70G-62/-37(Mut), 5′-GAGGAGGGAGCGttCGGGGCTCCATG-3′; CMHSP70G-114/-94(Mut), 5′-AACCCCTGcAACATTtCGGTC-3′; CMHSP70G-204/-172(Mut), 5′-GAACCCCAcAAGCGTtTGcAGAGTTtTGGGGAG-3′. EMSA was carried out as described^9^.

### ChIP analysis

ChIP was carried out as described previously^30^. All ChIP experiments were performed at least three times on independent chromatin preparations. Normal rabbit IgG (sc-2027) was purchased from Santa Cruz Biotechnology. The antibodies used were anti-Sp1 (07-645, Millipore), anti-HSF1 (#4356, Cell Signal Technology), and anti-Histone H3 (ab1791, Abcam). PCR was performed with the following set of primers: CMHSP70G-403F, 5′-ACACCAGAACTCTTTCTGACTGC-3′, and CMHSP70G-18R, 5′-CTCGGGCTTTATACGTCTCCAT-3′, with which the amplicon size was 386 bp. The PCR products were fractionated using a 2% agarose gel. ChIP quantitative PCR (ChIP-qPCR) was performed using the KOD SYBR qPCR Mix (Toyobo) with the same primer set. The anti-Sp1 and anti-HSF1 antibodies were validated for immunoblotting and immunoprecipitation using the corresponding *in vitro-*translated chipmunk proteins (Supplementary Fig. S12).

### Immunoblot analysis

Nuclear extracts and whole cell extracts were prepared using NUM buffer as described in Reinke *et al*.^18^ or Nuclear/Cytosol Fractionation Kit (BioVision). Immunoblotting was carried out as described previously^31^. The antibodies used were anti-Sp1 (07-645, Millipore), anti-Actin (sc-8432) and anti-USF1 (sc-229, Santa Cruz Biotechnology), anti-HSF1 (#4356, Cell Signal Technology), and anti-HSP70 (SPA-812) and anti-HSC70/HSP70 (SPA-822, StressGen).

### Immunohistochemistry

Blocks of freshly dissected liver were embedded in FSC22 Blue compound (Leica Biosystems), frozen in liquid nitrogen, and stored at −80 °C until use. Frozen liver blocks were sectioned at a thickness of 7 μm with a Leica CM1850 Cryostat (Leica Biosystems) and placed on MAS-coated glass slides (Matsunami Glass). Sections were fixed with 10% Formaldehyde Neutral Buffer Solution (Nacalai Tesque) for 30 min at room temperature, and after being washed with PBS, were incubated with blocking buffer (2% milk, 0.5% Triton) for 1 h at room temperature. The sections were then incubated with anti-HSF1 polyclonal antibodies (1:1000 dilution) (#4356, Cell Signaling Technology) at 4 °C overnight. After being washed with 0.05% Triton X-100 in PBS for 60 min at room temperature, the sections were incubated with Alexa Fluor 488 goat anti-rabbit IgG antibody (A11008, Thermo Scientific) for 1 h at room temperature. After being washed with 0.05% Triton X-100 in PBS for 60 min at room temperature, the sections were counterstained with 10 μg/ml Hoechst 33258 (Thermo Scientific) for 5 min at room temperature to detect nuclei. Finally, the sections were covered with 1 drop of VECTASHIELD Mounting Medium (Vector Laboratories), and visualized with a BZ-8100 fluorescence microscope (Keyence).

### Primary hepatocyte culture and siRNA transfection

Primary hepatocytes were obtained from chipmunk liver by the collagenase method. After liver perfusion with collagenase type IV (50 mg/100 ml) (Worthington), the dispersed cells were filtered successively through 500-μm and 125-μm stainless-steel sieves, and washed with DMEM four times with centrifugation at 70 × g for 3 min at 4 °C. The final hepatocyte pellet was resuspended in Williams’ medium E supplemented with 10% FCS, 1 μM insulin, 1 μM dexamethasone, 10^−11^ M Hepatocyte growth factor (HGF), and 10^−9^ M Epidermal growth factor (EGF). The siRNA duplexes targeting chipmunk *HSF1* were purchased from B-Bridge International. The sense sequences of siRNAs were as follows: HSF1-479, 5′-CCAUGAAGCACGAGAACGA(dTdT)-3′; HSF1-1367, 5′-GGAAGCAGCUGGUGCAGUA(dTdT)-3′. The siRNA duplex targeting Jellyfish *GFP* was purchased from Nippon Gene and used as a negative control. Primary hepatocytes were transfected with siRNA by Lipofectamine RNAiMax (Invitrogen) using a reverse-transfection protocol (Invitrogen). In 60-mm Biocoat collage I culture dishes (Corning), 60 pmol siRNA was diluted with 1 μl Opti-MEM I medium (Invitrogen), and then mixed with 10 μl Lipofectamine RNAiMax. After 20 min, 1 × 10^6^ hepatocytes were added to each plate, and incubated at 37 °C in a CO_2_ incubator. After 16 h, the medium was changed to Williams’ medium E supplemented with 10% FCS, 1 μM insulin, 1 μM dexamethasone, 10^−11^ M HGF, 10^−9^ M EGF, 100 U/ml penicillin, 0.1 mg/ml streptomycin, 0.5 μg/ml fungizone, and 0.1 mg/ml kanamycin. Hepatocytes were cultured for another 2 days.

### RT-qPCR

Total RNA was prepared from chipmunk hepatocytes using the RNeasy Mini Kit (Qiagen), according to the manufacturer’s instructions. First-strand cDNA was synthesized with the PrimeScript 1st strand cDNA Synthesis Kit (Takara Bio). qPCR was carried out using KOD SYBR qPCR Mix (Toyobo) with the following primer sets: CMALB-816F, 5′-AGATGGCAACAGATCTTACCAAA-3′, and CMALB-1369R, 5′-CTGGGGTGTTTTCTGAGTGTAAC-3′; CMGAPDH-1F, 5′-GGGTGTGAACCATGAGAAGTATG-3′, and CMGAPDH-1R, 5′-ACAGTCTTCTGAGTGGCAGTGAT-3′; CMHSF1-490F, 5′-GGAGAACATCAAGAGGAAAGTGA-3′, and CMHSF1-738R, 5′-AGATCAGGAACTGAATGAGCTTG-3′; CMHSP70-2149F, 5′-CTGTTTGCCCTCTGTTGTTATCT-3′, and CMHSP70-2403R, 5′-AAACAATATTTCCCCGATTTTGT-3′.

### Statistical Analysis

Kolmogorov-Smirnov test, Student’s *t* test (2-tailed), Welch’s *t* test (2-tailed), Wilcoxon rank sum test, Tukey multiple comparison test, one-way ANOVA, or two-way ANOVA was used to compare the difference. Student’s *t* test (in case of homogeneity of variances) or Welch’s *t* test (in case of heteroscedasticity) served to compare two values. Variance analysis was tested by *F* test at a 5% probability level for significance. When the interaction was significant at p < 0.05 by one-way ANOVA or two-way ANOVA, post hoc multiple comparison was performed using the Tukey multiple comparison test. Two-way ANOVA was also used to analyze the interaction between treatments. All the data in this paper were confirmed to be normally distributed by a Kolmogorov-Smirnov test, even when the data analyzed by Wilcoxon rank sum test. Student’s *t* test and Welch’s *t* test were performed in excel workbook, and Kolmogorov-Smirnov test, Wilcoxon rank sum test, Tukey multiple comparison test, and one-way or two-way ANOVA were performed in R software.

## Supplementary information

Supplementary Figures

## Data Availability

The complete DNA sequence data of the chipmunk have been submitted to the DDBJ. The DDBJ accession numbers are LC388388 and LC388389 (albumin), LC388390 (hsp70), LC388391 (hsf1), LC388392 (gapdh), and LC388393 (sp1). All data generated or analyzed during this study are included in this article and its Supplementary Information file.
