# Supplementary material for: Circadian transcription factor HSF1 regulates differential HSP70 gene transcription during the arousal-torpor cycle in mammalian hibernation
Source: Sci Rep. 2019 Jan 29;9:832. doi: 10.1038/s41598-018-37022-7 (PMC6351659; doi:10.1038/s41598-018-37022-7)
Supplement: Supplementary file 1 — Supplementary Figures [file 41598_2018_37022_MOESM1_ESM.pdf]

Supplementary information

Circadian transcription factor HSF1 regulates differential *HSP70* gene transcription during the arousal-torpor cycle in mammalian hibernation

Daisuke Tsukamoto,[1,*] Tomoko Hasegawa,[1] Shin-ichi Hirose,[1] Yukina Sakurai,[1] Michihiko Ito,[1] and Nobuhiko Takamatsu[1,*]

[1]Laboratory of Molecular Biology, Department of Biosciences, School of Science, Kitasato University, 1-15-1 Kitasato, Minami-ku, Sagamihara, Kanagawa 252-0373, Japan.

*Corresponding authors: tsukamot@kitasato-u.ac.jp (D.T.), takamatu@sci.kitasato-u.ac.jp (N.T.)

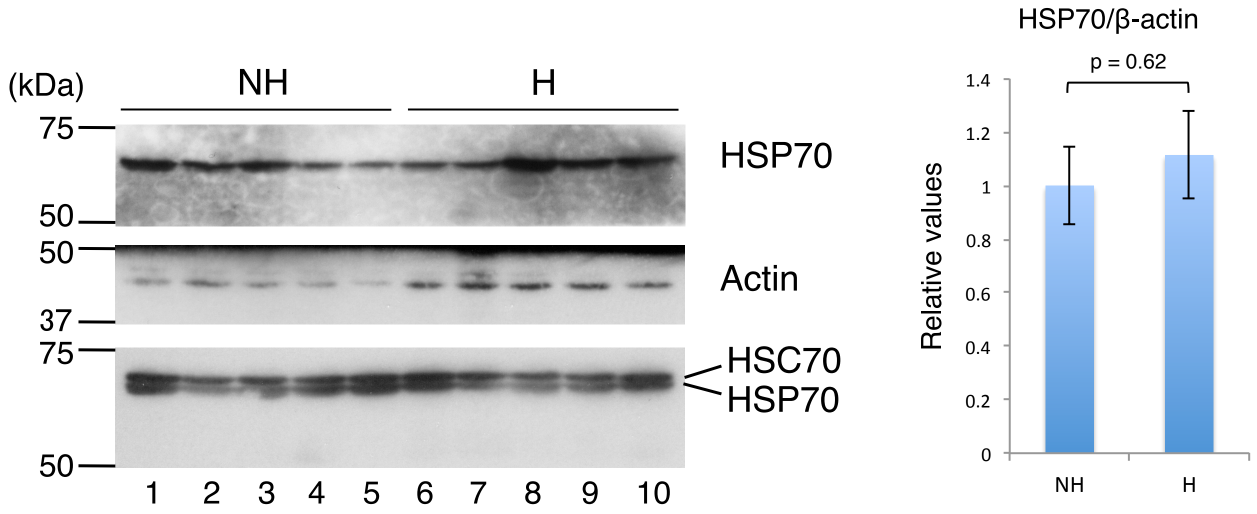

Supplementary Fig. S1

Immunoblot analysis of HSP70 and actin using whole cell extracts prepared from the liver of the same chipmunks as in Fig. 1 (left). Antibodies against HSP70 (StressGen, SPA-812) (left top panel), Actin (SantaCruz, sc-8432) (left middle panel), and HSC70/HSP70 (StressGen, SPA-822) (left bottom panel) were used by immunoblot analysis. Signals were quantified using ImageJ. HSP70 values (left top panel) were normalized to those of actin, and results are shown relative to the value obtained for nonhibernating chipmunks (NH) (right). Results are shown as means ± SEM. Student's *t*-test was used to assess the difference.

```
acaacacaag cctaaagctg gcccttgcgg tcacttcaca ctacggggct ggccgccagc -301

aaagccatgg agacggaggc gacagtcacc tccccacccc accccacacc cccctccgca -241

ctcgccctcc cctcggggcc ccctatcccc tccagtgaac cccagaagcg tctggagagt -181
                                                     HSE-B
tctggggagg ggcggcaccc tggcctctga ttggtgccgg gaggccaggg gcagaacgcc -121

aggagaaacc cctggaacat tccggtcccg acagcgccat ccggcacggc gattggccga  -61
               HSE-A                                    CCAAT
ggagggagcg ggcggggctc catggagacg tataaagccc gagcgggctg cgcggtgagg   -1
           GC                      TATA
AACAGCGAGC CTCAGCAGCT GCGGACGCAG GCGCAGGTTC TGCGTTCCCT GAGCGCACAC  +60

CGGAGCGGTC GGCTTCACAC TCTCATCCAG GACCGAGCTT CTCGTCGCGG GTCGCGTCCG +120

CCCACCGCAG CCAGAACCTC AGCGCGGATC CCAGAGAGCG GGCACCGAC ATGGCCAAAG +180
                                                        M  A  K
```

Supplementary Fig. S2

Nucleotide sequence of the proximal region of the chipmunk *HSP70* gene. The transcription start site is indicated as +1. Exon sequences are shown in upper-case letters.

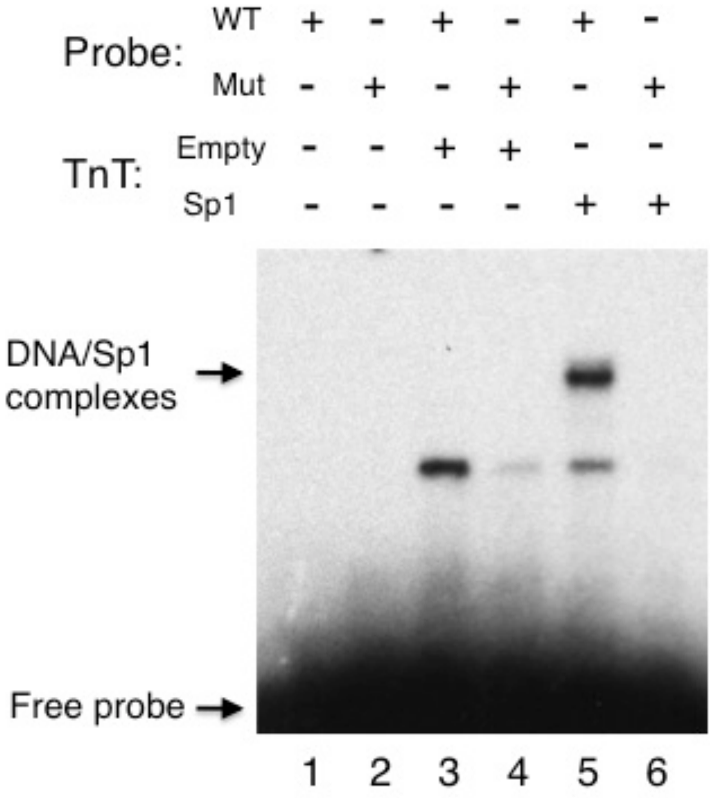

Supplementary Fig. S3

^{32}P-labeled CMHSP70G-62/-37(WT) (lanes 1, 3 and 5) or CMHSP70G-62/-37(Mut) (lanes 2, 4 and 6) was incubated with the *in vitro* transcription-translation products of pcDNA3 (Empty vector: lanes 3 and 4) or pcDNA3/mSp1 (lanes 5 and 6), and the DNA-protein complexes were separated by electrophoresis in a 4% polyacrylamide gel.

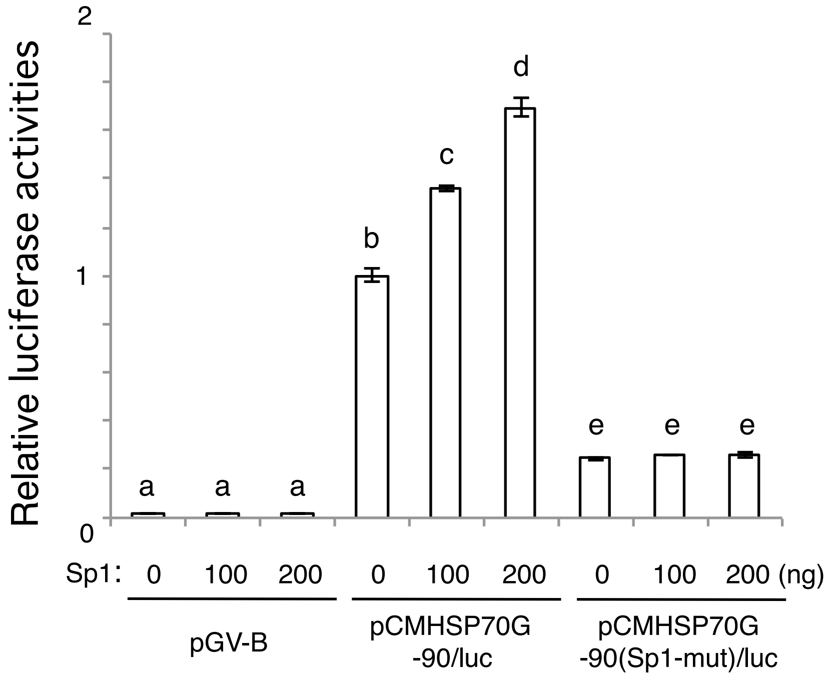

Supplementary Fig. S4

HepG2 cells were transfected with the indicated amounts of pcDNA3/mSp1 together with an *HSP70* gene promoter-reporter construct [pCMHSP70G-90/luc or pCMHSP70G-90(Sp1-mut)/luc], and pRL-ALB, which contains the promoter region of the chipmunk albumin gene upstream of the *Renilla* luciferase coding region. The firefly luciferase activity was normalized to the *Renilla* luciferase activity, and the data are shown relative to the activity obtained with pCMHSP70G-90/luc alone. Data represent means ± SEM of a representative experiment performed in quadruplicate. Experiments were repeated three times. Different letters (a-e) indicate significantly different values at $p < 0.05$; two-way ANOVA with Tukey-Kramer post hoc test.

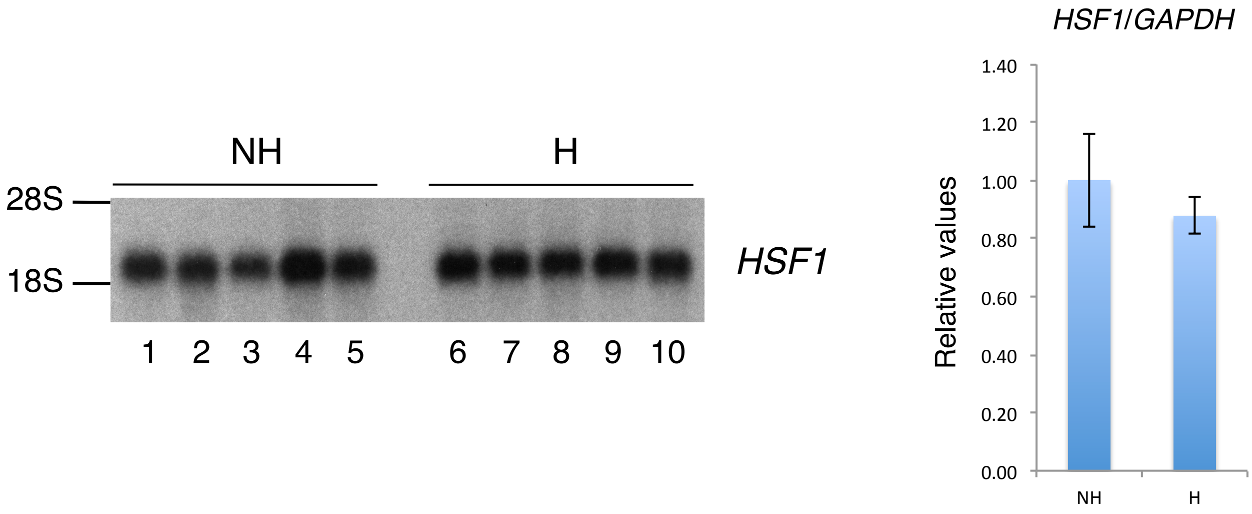

Supplementary Fig. S5

Northern blot analysis (left panel) and RT-qPCR (right panel) were performed to examine the *HSF1* mRNA level using the same liver poly(A)$^{+}$-RNA as in Fig. 1. In the RT-qPCR analysis, the *HSF1* mRNA levels were normalized to that of the *GAPDH* mRNA (Fig. 1), and the results are shown relative to the value obtained for nonhibernating chipmunks (NH). Results are shown as means ± SEM. The difference was not significant ($p>0.05$); Student's *t*-test.

a

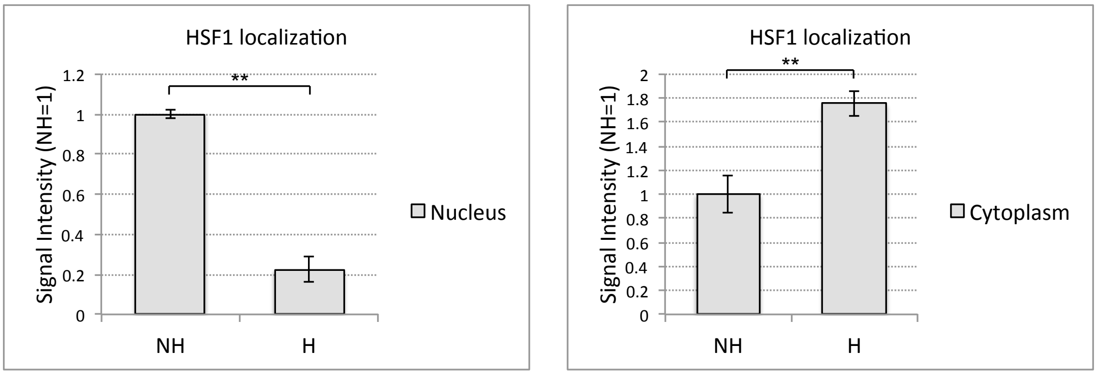

b

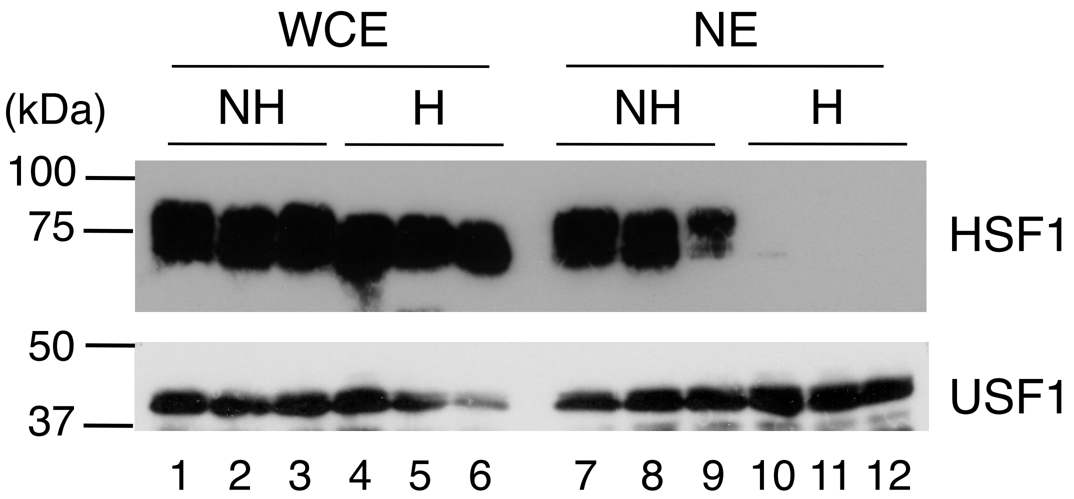

Supplementary Fig. S6

(a) The fluorescence signal quantification of immunohistochemistry in Fig. 3c. The signal intensities of eight cells chosen at random were analyzed with Image Studio 4.0 software (LI-COR Biosciences UK Ltd, Cambridge, UK) and normalized with the Hoechst signal of the same eight cells. Results are means ± SEM. Welch's *t* test (unpaired, 2-sided) was used to compare the difference. (b) Immunoblot analysis of HSF1 and USF1 was performed using whole cell extracts (WCE) or nuclear extracts (NE) prepared from the kidney of three each of nonhibernating (NH) and hibernating (H) chipmunks.

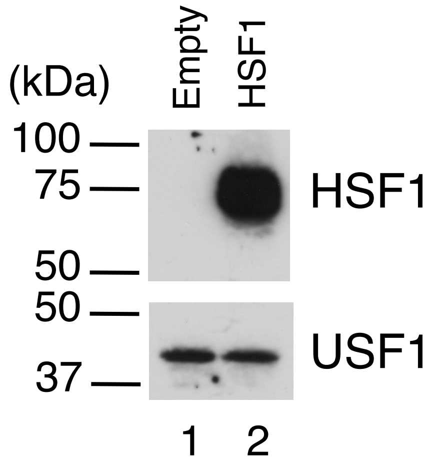

Supplementary Fig. S7

Primary hepatocytes prepared from a hibernating chipmunk were transfected with pcDNA3 (Empty) or pcDNA3/mHSF1, and the overexpression of HSF1 was confirmed by immunoblot analysis of nuclear extracts.

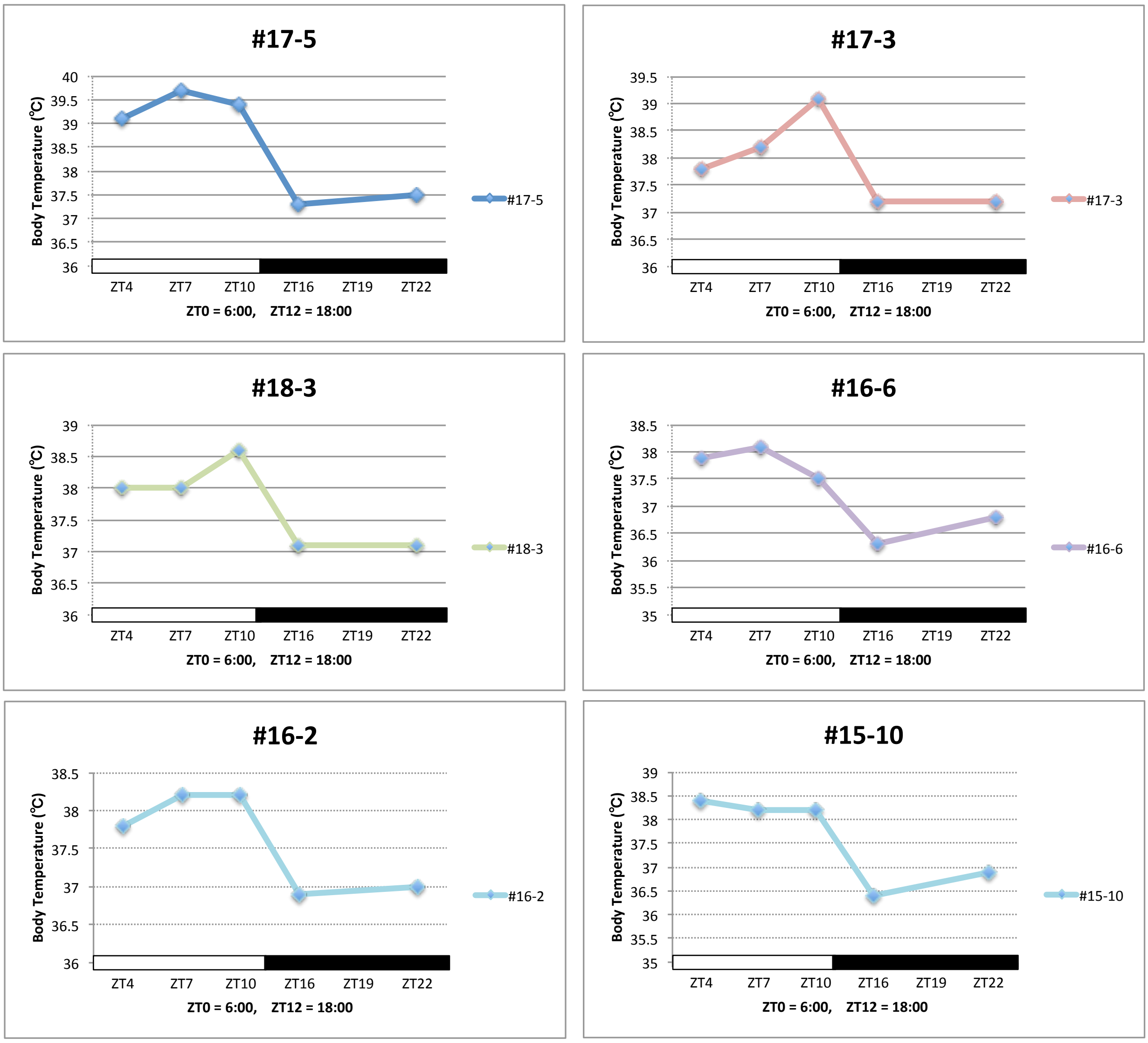

Supplementary Fig. S8

Body temperatures (Tb) of nonhibernating chipmunks were measured by a thermistor probe. Six male chipmunks (#17-5, #17-3, #18-3, #16-6, #16-2, and #15-10) were individually housed and provided with standard rodent chow and water *ad libitum*. They were kept at 23°C with a 12h:12h light (white box):dark (black box) photoperiod {light on at 6 AM (ZT0)} during the nonhibernation season (April-September). Chipmunks' rectal temperatures were measured by a thermistor probe at the indicated time points.

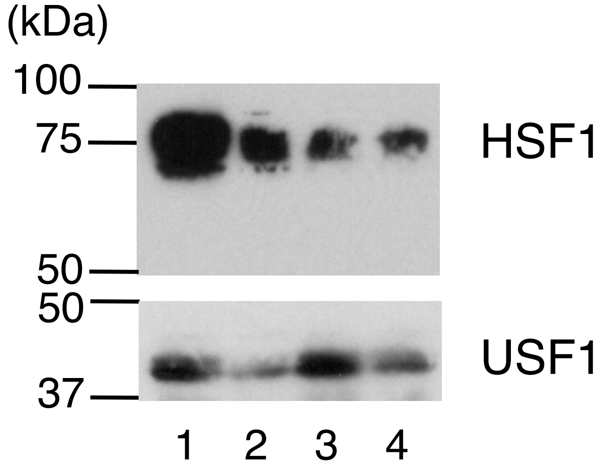

Supplementary Fig. S9

HSF1 activation by Tb rhythms additional data related to Fig. 5a. Immunoblot analysis of HSF1 and USF1 using nuclear extracts prepared from the liver of nonhibernating chipmunks different from the samples used for Fig. 5a.

Animal ID	Condition	Tb	Measuring time point
#16-20	IBA	24℃	1 hr after arousal
#16-5	IBA	29℃	1 hr after arousal
#16-9	IBA	34.8℃	2 hr after arousal
#18-2	IBA	36.5℃	4 hr after arousal
#16-12	IBA	35.8℃	5 hr after arousal
#16-3	IBA	36.8℃	6-7 hr after arousal
#17-6	H	6.5℃	2 d after entry into torpor
#17-2	H	6.3℃	3 d after entry into torpor
#17-4	H	6.2℃	4 d after entry into torpor

Supplementary Fig. S10

Body temperatures (Tb) of chipmunks during an interbout arousal (IBA) and a torpor bout (H) were measured by a thermistor probe. Nine male chipmunks (#16-20, #16-5, #16-9, #18-2, #16-12, #16-3, #17-6, #17-2, and #17-4) were kept under a constant condition of 5°C in darkness during the hibernation season (October-March). The conditions of hibernating chipmunks (weight, 90-110 g; age, 1-3 years) were monitored by an infrared activity sensor, and the sampling time of hibernating chipmunks were 2-4 days after entry into deep torpor. The interbout awake chipmunks (weight, 90-110 g; age, 1-3 years) were obtained approximately two to four months after the first entry into torpor between December and February. Chipmunks' rectal temperatures were measured by a thermistor probe at the indicated time points.

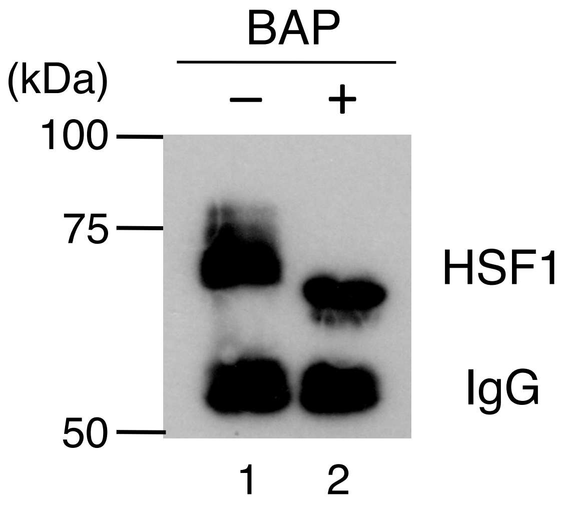

Supplementary Fig. S11

HSF1 was immunoprecipitated from the liver nuclear extract of a nonhibernating chipmunk, treated with bacterial alkaline phosphatase (+) (Toyobo), and then subjected to immunoblot analysis.

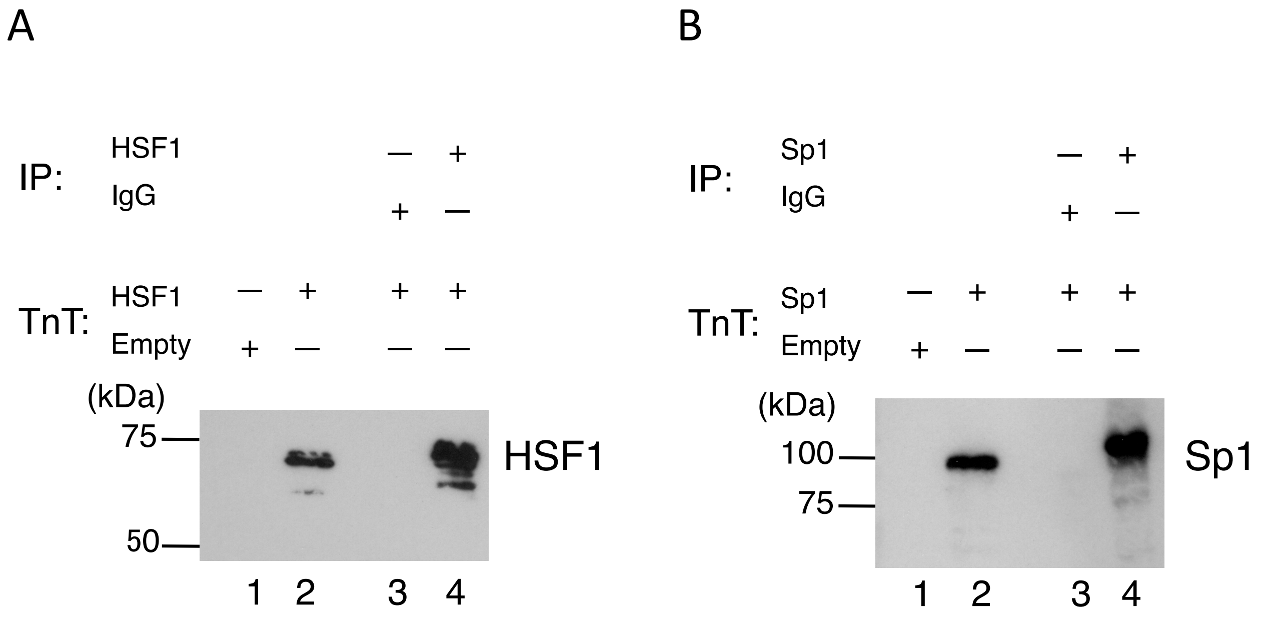

Supplementary Fig. S12

Antibodies against HSF1 (**A**) and Sp1 (**B**) were evaluated by immunoblot analysis and immunoprecipitation (IP) using *in vitro*-translated chipmunk HSF1 and Sp1.

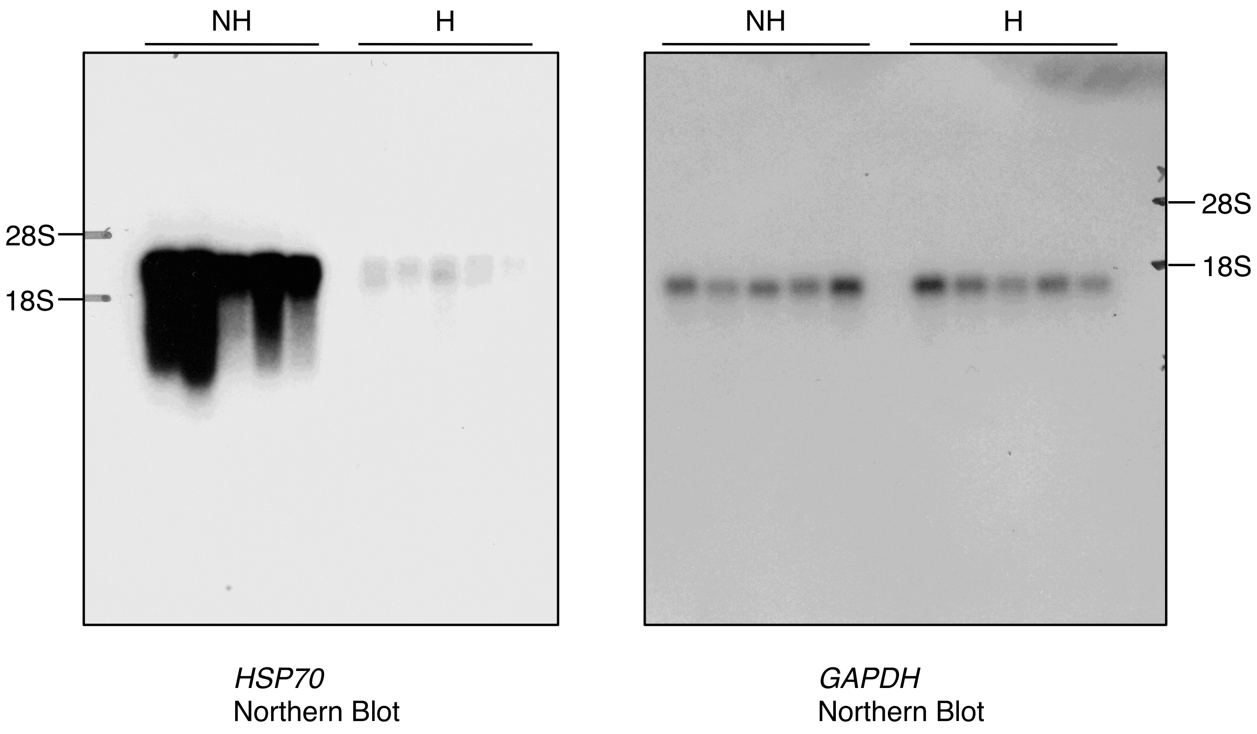

Supplementary Fig. S13

Full-length images of northern blots from Fig. 1.

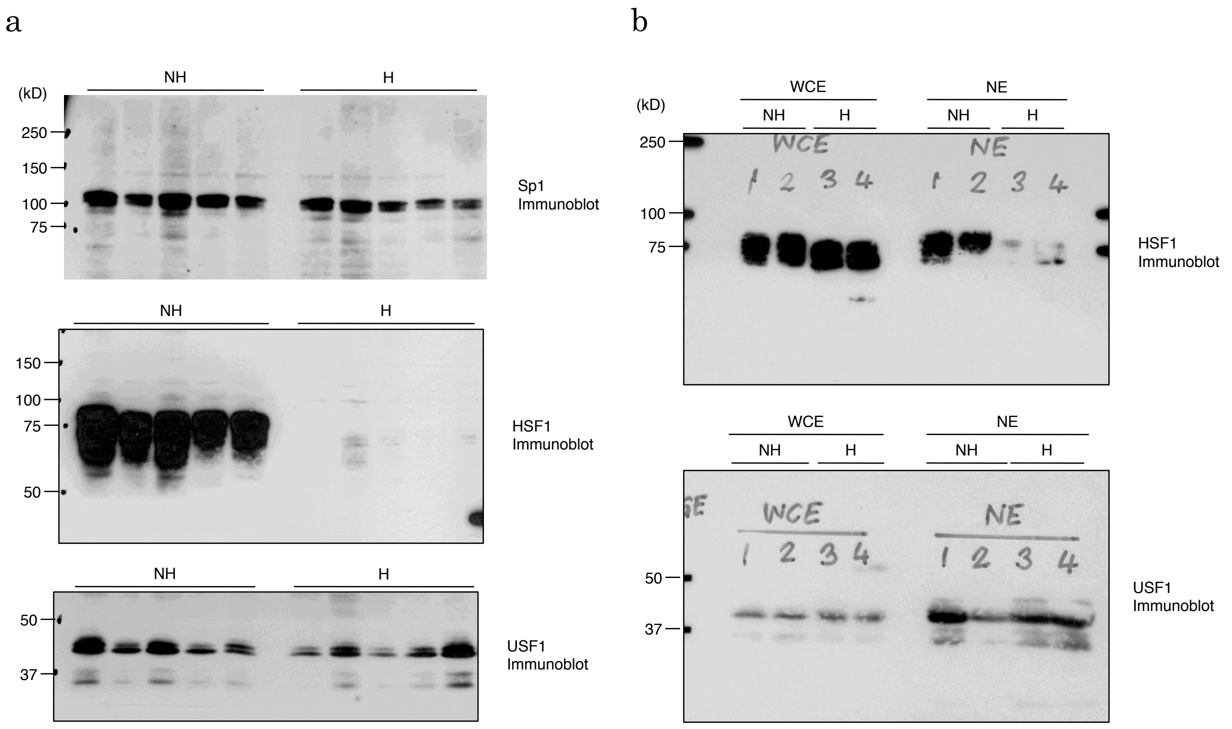

Supplementary Fig. S14

Full-length images of immunoblots from Fig. 3a-b.

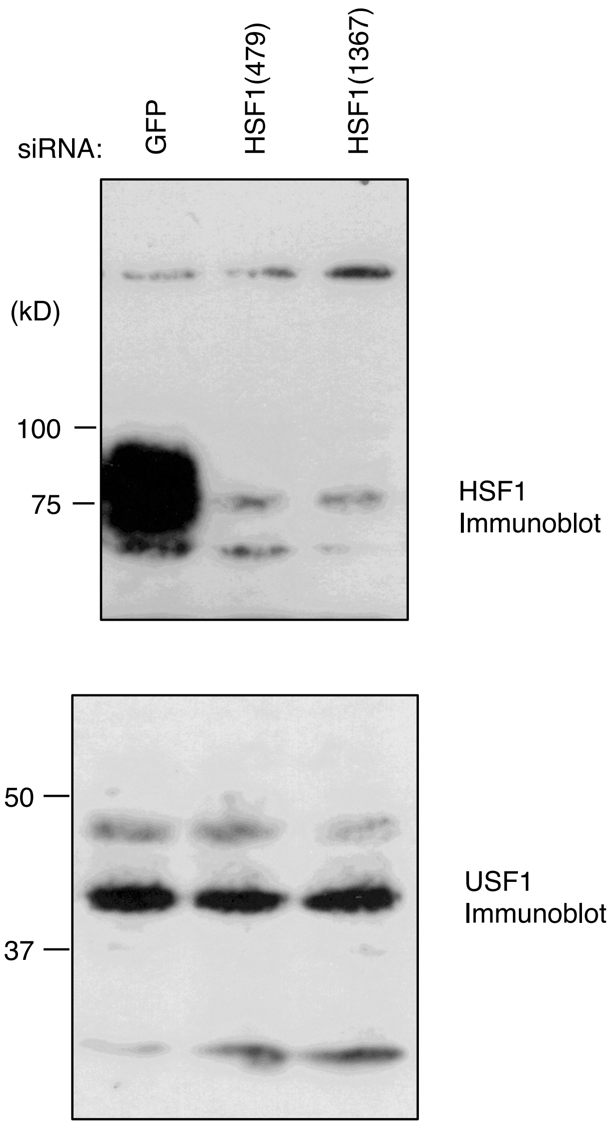

Supplementary Fig. S15

Full-length images of immunoblots from Fig. 4b.

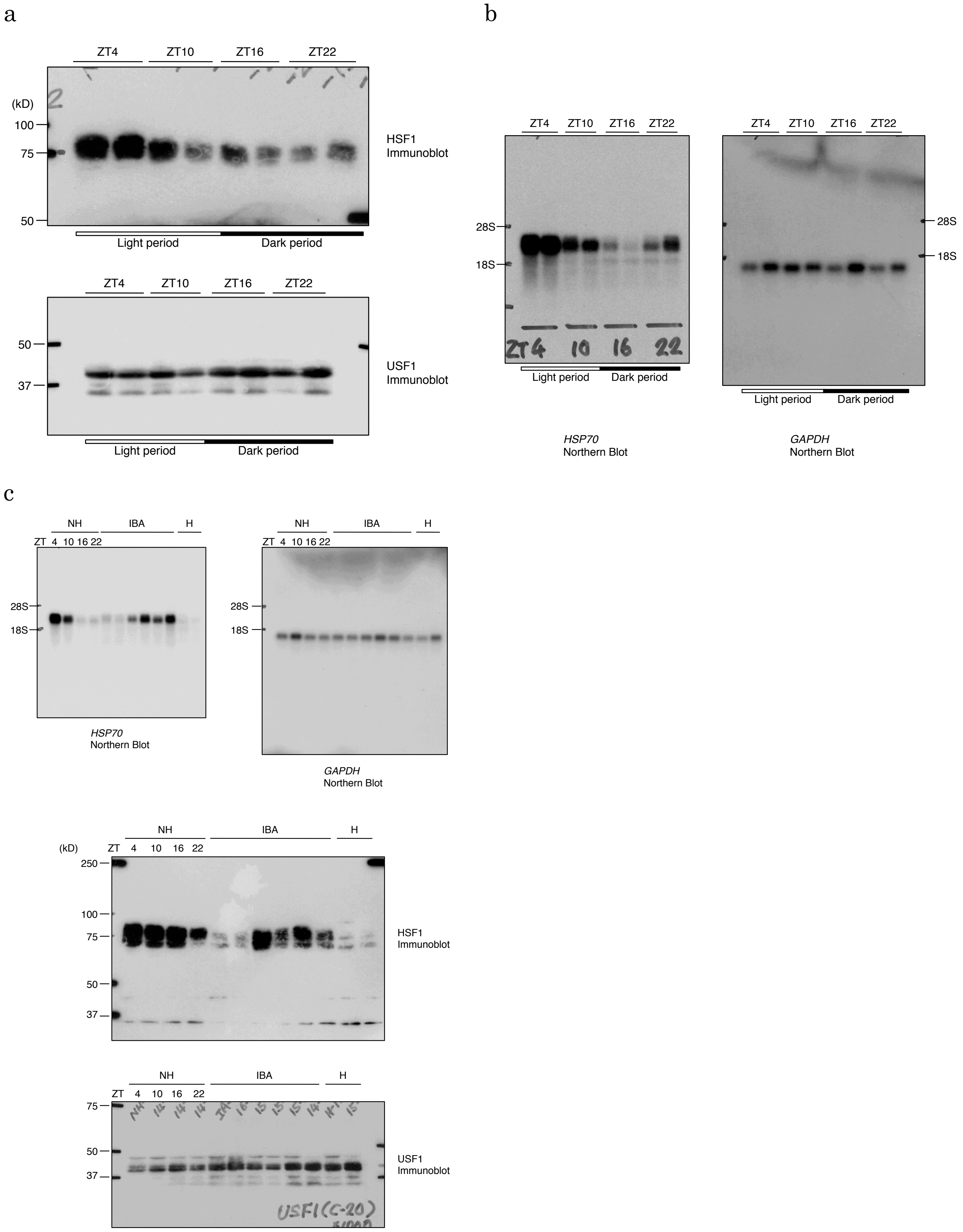

Supplementary Fig. S16

Full-length images of immunoblots and northern blots from Fig. 5a-c.
